# The Undiagnosed Chronically-Infected HCV Population in France. Implications for Expanded Testing Recommendations in 2014

**DOI:** 10.1371/journal.pone.0126920

**Published:** 2015-05-11

**Authors:** Cécile Brouard, Yann Le Strat, Christine Larsen, Marie Jauffret-Roustide, Florence Lot, Josiane Pillonel

**Affiliations:** 1 Infectious Diseases Department, French Institute for Public Health Surveillance (InVS), Saint-Maurice, France; 2 Cermes3 (Inserm U988/CNRS UMR8211/EHESS/Université Paris-Descartes), Paris, France; University of Modena & Reggio Emilia, ITALY

## Abstract

**Background:**

Recent HCV therapeutic advances make effective screening crucial for potential HCV eradication. To identify the target population for a possible population-based screening strategy to complement current risk-based testing in France, we aimed to estimate the number of adults with undiagnosed chronic HCV infection and age and gender distribution at two time points: 2004 and 2014.

**Methods:**

A model taking into account mortality, HCV incidence and diagnosis rates was applied to the 2004 national seroprevalence survey.

**Results:**

In 2014, an estimated 74,102 individuals aged 18 to 80 were undiagnosed for chronic HCV infection (plausible interval: 64,920-83,283) compared with 100,868 [95%CI: 58,534-143,202] in 2004. Men aged 18-59 represented approximately half of the undiagnosed population in 2014. The proportion of undiagnosed individuals in 2004 (43%) varied from 21.9% to 74.1% in the 1945-1965 and 1924-1944 birth cohorts. Consequently, age and gender distributions between the chronically-infected (diagnosed and undiagnosed) and undiagnosed HCV populations were different, the 1945-1965 birth cohort representing 48.9% and 24.7%, respectively.

**Conclusions:**

Many individuals were still undiagnosed in 2014 despite a marked reduction with respect to 2004. The present work contributed to the 2014 recommendation of a new French complementary screening strategy, consisting in one-time simultaneous HCV, HBV and HIV testing in men aged 18-60. Further studies are needed to assess the cost-effectiveness and feasibility of such a strategy. We also demonstrated that data on the undiagnosed HCV population are crucial to help adapt testing strategies, as the features of the chronically-infected HCV population are very distinct.

## Introduction

In the last decade, the management of Hepatitis C Virus (HCV) infection has significantly evolved. The greatest advance concerns the development of direct-acting antiviral agents (DAAs) which have revolutionized HCV treatment. Ongoing clinical trials demonstrate sustained virologic response rates exceeding 90% and minimal side effects with interferon-free all-oral combinations [1,2]. These DAAs may improve HCV treatment adherence and compliance provided that they are made widely available despite their projected high costs. Treatment as a prevention strategy could then become feasible in injecting drug users (IDUs) who constitute the main reservoir of HCV transmission in western countries. A marked reduction of HCV prevalence over the medium-term could be possible if even only a small proportion of IDUs were treated [3–5].

Improving care for HCV-infected patients also involves the development and widespread use of non-invasive methods to assess liver fibrosis, including the measurement of serum biomarkers and of liver stiffness using elastography. These two methods are now recommended for first-line evaluation of liver fibrosis in patients with chronic hepatitis C [6]. By reducing the need for liver biopsy, they improve linkage to care and therefore treatment initiation.

New screening tools are also available upstream of hepatitis C management. Rapid diagnostic tests using non-traditional biological matrices, such as gingival crevicular fluid and total capillary blood, are rapidly evolving and present satisfactory diagnostic accuracy [7]. By providing patients with testing opportunities in locations other than biological laboratories (point-of-care testing), rapid diagnostic tests help extend first-line HCV screening, especially towards the hard-to-reach populations including IDUs, migrants and those with a low socio-economic status [8].

In this context, the eradication of HCV infection would seem to be achievable since the disease meets most scientific and socio-political criteria for disease eradicability [3,9]. However, eradication would not be possible without a significant improvement in screening effectiveness [10]. Indeed, HCV infection is under-diagnosed since HCV infection is often asymptomatic for many years. In the United States (US), it has been estimated that almost half of the HCV-infected population were unaware of their status in the 2001–2008 period [11]. This under-diagnosis constitutes a missed opportunity for infected individuals to benefit from early access to care and therefore to limit their risk of morbidity and mortality. At the group level, under-diagnosis limits a general reduction in viral transmission. To improve the identification of people living with chronic HCV infection, some western countries, like the U.S. and Canada, have recently implemented expanded HCV screening recommendations for one-time testing in specific birth cohorts in addition to existing risk-based strategies [12,13]. This population-based testing strategy concerns the birth cohort of people born between 1945 and 1965 in the US, and between 1945 and 1975 in Canada, because these age-groups have the highest HCV antibodies prevalence [12,13].

Complementing existing risk-based testing strategies [14] with additional population-based screening is currently a topical issue also in France. Indeed, despite the country providing some of the best hepatitis care and highest screening rates [15–17] in Europe—most probably the result of three national action plans for prevention implemented by the French Ministry of Health since 1999 [18]—several indicators demonstrate that there is still a great deal of room for improvement. First, only 57% of chronically-infected HCV patients in France were aware of their status in 2004 [19]. Second, among diagnosed people, less than 20% were diagnosed for risk exposure, the majority (57%) being diagnosed during systematic screening (i.e. during a check-up, blood donation, etc.) [20,21]. The high proportion of patients with cirrhosis or hepatocellular carcinoma at the time of HCV diagnosis (12%) also suggests that there are limitations with using the risk-based testing strategy [20]. HCV screening effectiveness in France, just as in the US and Canada, may be improved by complementing current risk-based testing with population-based testing. To identify the population to be targeted (age-group and/or gender), data on the characteristics of undiagnosed persons are essential.

Accordingly, this work aimed to estimate the number of adults with undiagnosed chronic HCV infection in France in 2014 and their age and gender distributions in order to help adapt existing testing strategies. The secondary objective was to study the differences between these estimates and those for 2004. This research was conducted in the context of the first French report for recommendations on the management of patients infected with hepatitis C or B virus, published in May 2014 [22].

## Materials and Methods

We defined undiagnosed chronically-infected HCV individuals as persons with positive HCV RNA unaware of their infection. The number, proportion, and demographic distribution of these individuals were estimated from the most recent national seroprevalence survey conducted in 2004 [19] and updated for 2014 (except the proportion).

### 2004 seroprevalence data

A cross-sectional survey among a random sample of residents of mainland France, aged 18–80 years old, was conducted in 2004 to estimate the prevalence of hepatitis B and C virus infections [19]. Selected individuals received an invitation letter for a free medical checkup. Overall, 14,416 people were included after written informed consent. An anti-HCV antibody screening test was first carried out for all participants. HCV RNA detection was then performed for those individuals diagnosed anti-HCV positive. Data were collected for demographic characteristics, potential exposure to HCV and any prior HCV screening and results. Awareness of HCV infection was assessed using these two last variables.

### 2014 model-based estimates

**Fig 1:**
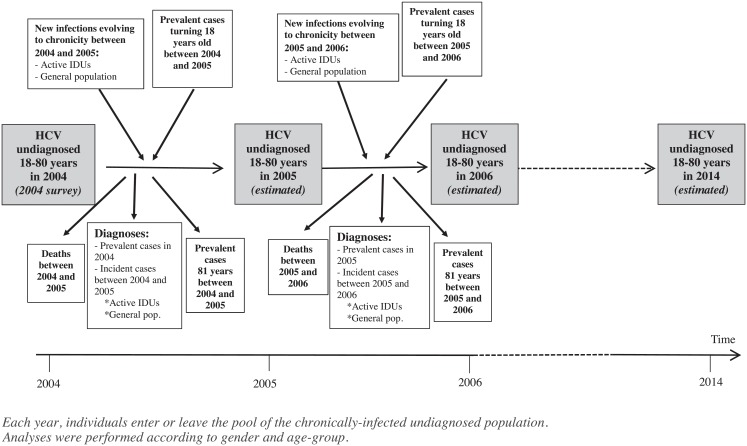
Chart flow of the model.

An epidemiologic model was applied to undiagnosed chronically-infected HCV cases aged 18 to 80 in 2004 to estimate the number of undiagnosed persons aged 18–80, and their age and gender distribution in 2014. Analyses were performed for each year from 2004 to 2014, for each gender and age-group (18–29, 30–39, 40–49, 50–59, 60–69 and 70–80) (Fig 1). Each year, new individuals entered in the pool of the undiagnosed chronically-infected population either because they were new infections evolving to chronicity or because they were prevalent undiagnosed cases turning 18. There were also outgoing cases from the pool of the undiagnosed chronically-infected population for three reasons: i) diagnoses between 2004 and 2014 of prevalent cases in 2004 (cases already infected in 2004) and incident cases over the period; ii) deaths; iii) undiagnosed cases turning 81.

#### Data

Multiple data sources were compiled to feed the model (Table 1). To include uncertainties of the model parameters, we studied several scenarios to estimate a plausible interval around the estimated number of undiagnosed HCV persons in 2014.

**Table 1:** Parameter values and data used for 2014 model-based estimates.

Parameters	Data	Estimates used for modelisation	References	Data availability
**New HCV infections evolving to chronicity among “active IDUs”**	Estimated number of French active IDUs in:			
	2006	81,000	[34]	
	2011	Low: 70,000; high: 90,000	Assumed from [35]	
	Age-and-gender distribution of French active IDUs in:			
	2004		[36]	See S1 Table
	2011		[37]	See S2 Table
	HCV seroprevalence among French active IDUs, by age-group and gender in:			
	2004		[36]	See S3 Table
	2011		[37]	See S4 Table
	HCV incidence in IDUs in western countries	Low: 6%, high: 18%	[23–30]	
	Rate of HCV spontaneous clearance	Low: 30%, high: 40%	[38,39]	
**New HCV infections evolving to chronicity among the “general population”**	Annual HCV incidence in repeat blood donors by age-group, gender in France		Method in [41]	See S5 Table
	Distribution of the French general population by age-group and gender		[40]	Available in [40]
	HCV seroprevalence in the French general population by age-group and gender		[19]	See S6 Table
	Rate of HCV spontaneous clearance	Low: 30%, high: 40%	[38,39]	
**Prevalent undiagnosed cases turning 18 between 2004 and 2014**	Number of chronically-infected undiagnosed HCV cases aged 18 by gender in 2004 in France	Men: 112, women: 122	[19]	
**Number of diagnoses among the HCV prevalent cases in general population in 2004**	Estimated proportion of chronically-infected HCV cases aware of their status in 2004 by age-group and gender		[19]	See Fig 2
	Assumption of an annual increase of 1% in the proportion of HCV cases aware of their status		[16]	Available in [16]
**Number of diagnoses among new HCV infections cases in general population between 2004 and 2014**	Estimated proportion of chronically-infected HCV cases aware of their status in 2004 among French general population		[19]	See Fig 2
	Distribution of the delay between at-risk exposure period and diagnosis of HCV cases for whom intravenous drug use and blood transfusion were not suspected	0–4 years: 20%, 5–10 years: 15%, > 10: 65%	[21]	
**Number of diagnoses among new HCV infections cases in active IDUs between 2004 and 2014**	Estimated proportion of anti-HCV IDUs aware of their status in:			
	2004	69%	[36]	
	2011	78%	[37]	
	Age at first injection in:			
	2004	20.4	[36]	
	2011	22	[37]	
	Distribution of the delay between at-risk exposure period and diagnosis of HCV cases for whom intravenous drug use was the suspected transmission mode	0–4 years: 47%, 5–10 years: 33%, > 10: 20%	[21]	
**Number of deaths**	Competitive mortality based on French life tables		[40]	Available in [40]
	Additional mortality for active IDUs incident cases between 2004 and 2014	5.27 for men, 9.74 for women	[42]	
**Undiagnosed HCV cases turning 81 between 2004 and 2014**	Exclusion of estimated undiagnosed cases aged over 80 in 2014	From 2014 model-based estimates		

ST: Supplemental table.

*New infections evolving to chronicity*: we assumed that new infections arose mainly among active IDUs (defined as people who injected drugs at least once during the previous month) and that a few cases occurred in the general population (not active IDUs). In the absence of accurate incidence rate estimates among IDUs in France, we reviewed the incidence rates from western countries, which varied from 2% to 33%, with a median and average value of 12% [23–30]. Since this parameter had a strong impact on the final estimate, we used a low estimate at 6% and a high estimate at 18%. We assumed that the incidence rate was 3.5 times higher for those aged under 30 years compared with those aged 30 or over [31–33]. We obtained the annual number of incident infections in active IDUs by multiplying the incidence rate by the estimated number of active French IDUs susceptible to HCV infection. The latter were assessed using: i) the number of active IDUs in France, estimated at 81,000 in 2006 by the French Monitoring Centre for Drugs and Drug Addiction [34], and assumed to range from 70,000 (low estimate) to 90,000 (high estimate) in 2011 [35]; ii) the age and gender distribution of active IDUs estimated through two editions of a national seroprevalence survey (ANRS-Coquelicot study), conducted in 2004 and 2011, among drug users enrolled in specialized services (S1 and S2 Tables) [36,37] and iii) HCV seroprevalence among active IDUs by age-group and gender in France (S3 and S4 Tables) [36,37]. Since our work focused on the chronically-infected HCV population, we subsequently removed the number of spontaneous clearances, estimated at 30% (low estimate) and 40% (high estimate) [38,39], from the estimated number of new HCV infections in active IDUs.

The number of new HCV infections evolving to chronicity in the general population was estimated in the same way using the HCV seroprevalence in the general population (S6 Table) [19], the age and gender distribution of the general population [40], the HCV incidence rate estimated in repeat blood donors (constituting a proxy of HCV incidence in the general population) (S5 Table) [41] and the rate of spontaneous viral clearance (between 30% and 40% [38,39]).

*Prevalent undiagnosed cases who turned 18 between 2004 and 2014*: this number was assessed using the estimated numbers of men and women aged of 18 undiagnosed for HCV chronic infection in 2004 [19].

*Diagnoses*: The number of prevalent undiagnosed cases in 2004 who were then diagnosed between 2004 and 2014 was estimated for each year, assuming that the proportion of chronically-infected HCV cases aware of their status in 2004 [19] increased by 1% per year [16]. Among incident cases in the general population, the yearly number of diagnosed cases was estimated using the proportion of chronically-infected HCV cases aware of their status in 2004 [19] and the distribution of the delays between infection and diagnosis of cases reported by hepatology wards in university hospitals to the national HCV surveillance system between 2004 and 2007 [21]. The estimated number of diagnoses of incident cases among active IDUs was based on the proportion of diagnosed among anti-HCV IDUs, on age at first injection of drugs [36,37] and on the distribution of the delay between infection and diagnosis for HCV cases for whom intravenous drug use was the suspected transmission mode [21].

*Deaths in prevalent and incident cases*: The competitive mortality rate was derived from the French life tables [40]. For active IDU incident cases, an excess mortality rate of 5.27 and 9.74, respectively, for men and women based on a French cohort study [42], was applied to competitive mortality values to take into account higher mortality than in the general population, due to IDU at-risk behaviors (overdose, suicide, road accidents, etc. [43]).

*Undiagnosed cases turning 81 between 2004 and 2014* were excluded from the final estimated number since our study population focused on the 18 to 80 year-old population.

Finally, eight scenarios combining low and high estimates of the number of active IDUs in 2011, HCV incidence rate in IDUs and HCV spontaneous clearance rate were studied (Table 2). From these scenarios, we obtained the final estimate (mid-point) and a plausible interval.

**Table 2:** Estimated numbers and distributions by gender and age-group of undiagnosed chronically-infected HCV population according to eight scenarios in 2014, mainland France.

				Midpoint estimate	Scenario 1	Scenario 2	Scenario 3	Scenario 4	Scenario 5	Scenario 6	Scenario 7	Scenario 8
**Parameters of sensitive analyses**	HCV incidence in IDUs				6%	6%	6%	6%	18%	18%	18%	18%
	Number of active IDUs in 2011				70,000	70,000	90,000	90,000	70,000	70,000	90,000	90,000
	HCV spontaneous clearance rate				40%	30%	40%	30%	40%	30%	40%	30%
**Data**	New infections evolving to chronicity				9,469	10,917	11,045	12,756	26,846	31,190	31,576	36,708
	Prevalent undiagnosed cases turning 18 years between 2004 and 2014				2,106	2,106	2,106	2,106	2,106	2,106	2,106	2,106
	Diagnoses				22,158	22,616	22,536	23,056	27,647	29,019	28,779	30,340
	Deaths				12,211	12,249	12,244	12,287	12,668	12,782	12,767	12,898
	Undiagnosed cases >80 years in 2014				13,140	13,140	13,140	13,140	13,140	13,140	13,140	13,140
**Estimates**	Undiagnosed chronically-infected HCV cases in 2014	Total		**74,102 (100%)**	64,920	65,872	66,086	67,233	76,345	79,201	79,844	83,283
		Men	18–39	**10,689 (14.4%)**	5,205	5,789	5,837	6,526	12,210	13,961	14,106	16,173
			40–59	**22,923 (30.9%)**	21,194	21,365	21,448	21,662	23,250	23,764	24,012	24,653
			60–80	**10,195 (13.8%)**	10,189	10,190	10,190	10,191	10,196	10,197	10,198	10,200
		Women	18–39	**3,453 (4.7%)**	1,865	2,023	2,094	2,290	3,765	4,240	4,452	5,042
			40–59	**6,495 (8.8%)**	6,120	6,159	6,171	6,217	6,579	6,694	6,730	6,870
			60–80	**20,346 (27.4%)**	20,346	20,346	20,346	20,346	20,346	20,346	20,346	20,346

Data analyses were performed using SUDAAN software (RTI International, Research Triangle Park, North Carolina), Stata 12.1 (Stata Corporation, College Station, TX) and Excel.

The protocol of the 2004 seroprevalence survey, that included collection of a written informed consent to participate in this study for all participants, was reviewed and approved by a national institutional and ethical review board (Comité consultatif de protection des personnes dans la recherche biomédicale, October 25, 2002, no. 02–035).

## Results

### 2004 seroprevalence data

**Fig 2:**
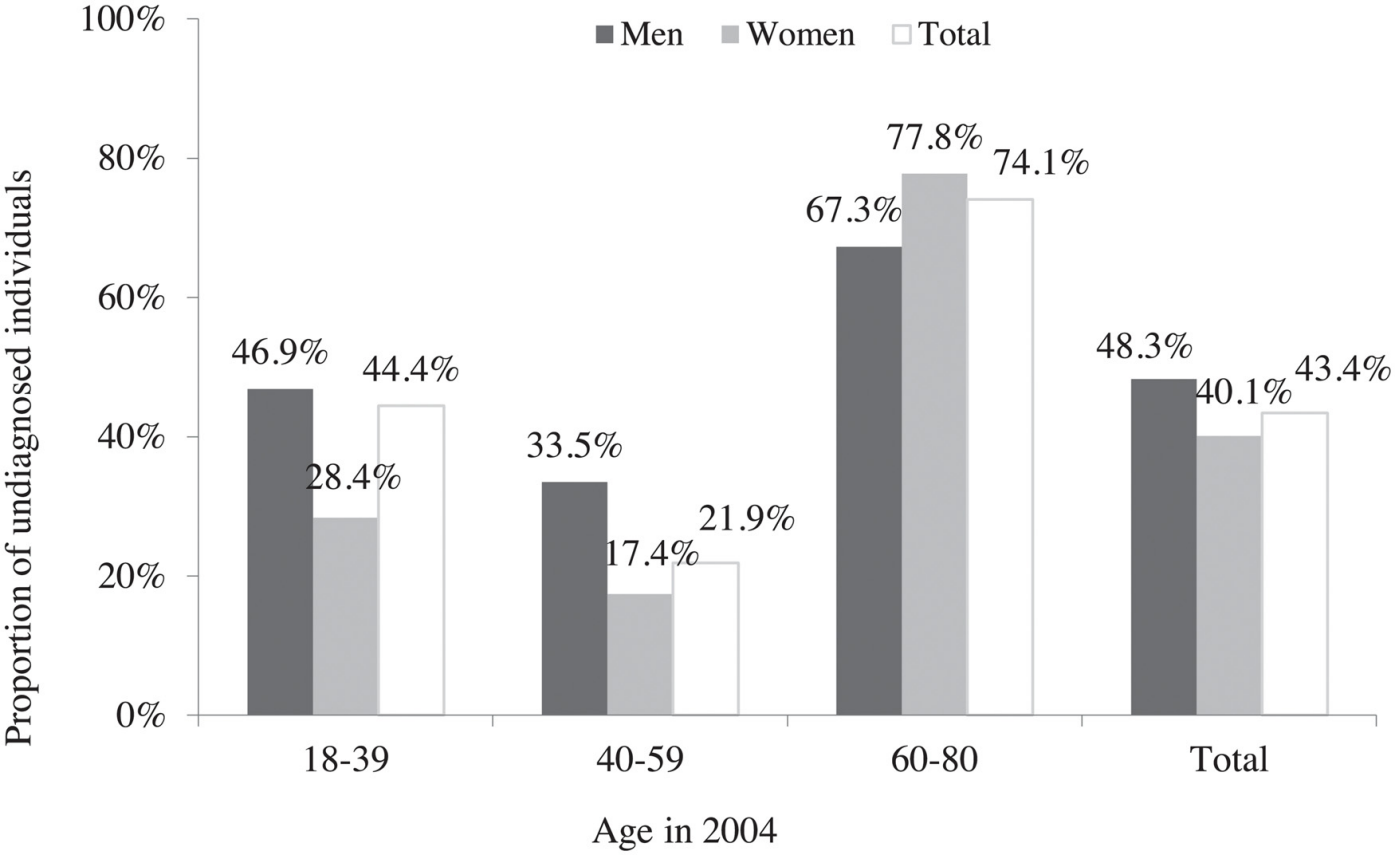
Estimated proportion of undiagnosed among chronically-infected HCV population by gender and age-group in 2004, mainland France.

Among 232,196 chronically-infected individuals with HCV [95%CI: 167,869–296,523] [19], we estimated that 100,868 [95%CI: 58,534–143,202] (43.4%) were undiagnosed in 2004. The proportion of undiagnosed individuals was higher in men than in women (48.3% *vs*. 40.1%, p<0.001). This figure was observed for the 18–39 and 40–59 year old age-groups (46.8% *vs*. 28.4%, p<0.001 and 33.5% *vs*. 17.4%, p<0.001, respectively) (Fig 2). Conversely, for the 60–80 year old age-group, we estimated that women were more frequently undiagnosed than men (77.8% *vs*. 67.3% respectively, p<0.001). For both genders, the 60–80 age-group (1924–1944 birth cohort) had the highest proportion of undiagnosed individuals (74.1%) while the 40–59 year old group (1945–1965 birth cohort) had the lowest (21.9%).

**Fig 3:**
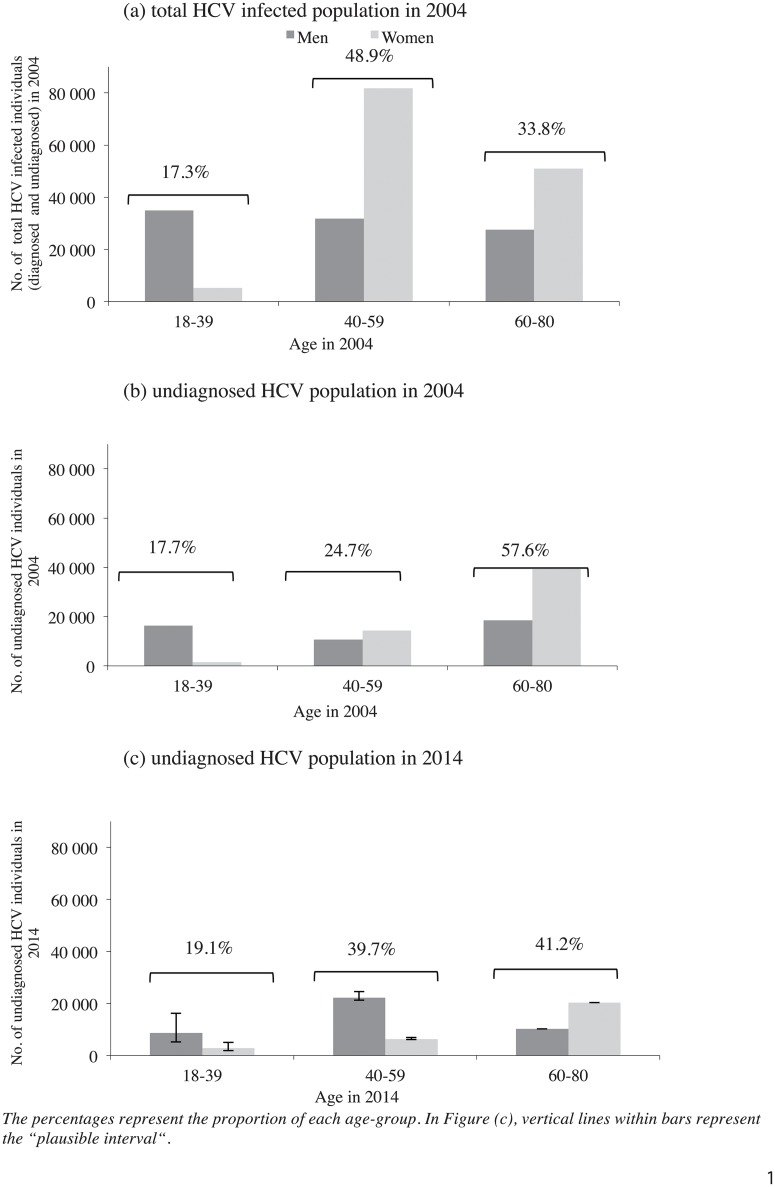
Age and gender estimated distributions of HCV chronically-infected population according to diagnosis and year, mainland France.

As a result, age and gender distributions between the chronically-infected (diagnosed and undiagnosed) HCV population and the undiagnosed population were quite different. Women represented 54.9% of the undiagnosed population *vs*. 59.5% of the infected population. Most of those undiagnosed were in the 60–80 year old age-group (1924–1944 birth cohort, 57.6%), the 40–59 (1945–1965 birth cohort) and 30–59 year old (1945–1975 birth cohort) age-groups representing, respectively, 24.7% and 41.0%. For the chronically-infected HCV population (Fig 3a and 3b) the latter two groups accounted for 48.9% and 63.7%, respectively.

Among the 100,868 chronically-infected undiagnosed persons in 2004, 32.4% reported having a blood transfusion before 1992 (the year when systematic anti-HCV screening in blood donations was introduced), 6.0% reported intravenous drug use at least once during their lifetime and 13.9% were born in moderate or high HCV endemic countries [44].

### 2014 model-based estimates

Over the 2004–2014 period, the estimated number of new infections evolving to chronicity varied according to the different scenarios studied, from 9,469 (scenario 1) to 36,708 (scenario 8). The estimated number of diagnoses rose from 22,158 to 30,340 and the estimated number of deaths from 12,211 to 12,898 (Table 2). The estimated number of prevalent cases who turned 18 years old and the number of cases who turned 81 in the period 2004–2014 were, respectively, 2,106 and 13,140, irrespective of the scenario.

In 2014, the estimated number of undiagnosed chronically-infected HCV individuals varied from 64,920 to 83,283 according to the scenario (plausible interval), with a midpoint estimate at 74,102, a decrease of 27% compared with 2004. The decline was particularly marked for the 60–80 age-group (-47%). In 2014, conversely to 2004, the undiagnosed population was mainly comprised of men (59.1%). The 60–80 year old age-group remained predominant (41.2%), especially the 70–80 that represented 36.9% of the undiagnosed (Fig 3c). In the undiagnosed population in 2014, there were few women under 60 years old whereas men aged 18 to 59 accounted for 45.4%.

## Discussion

To identify the population to be targeted by a possible population-based screening strategy as a complement to current risk-based testing in France, this work provides estimates for the numbers and age and gender distributions of the undiagnosed chronically-infected HCV population in mainland France. In 2014, an estimated 74,102 individuals aged 18 to 80 were undiagnosed for chronic HCV infection (plausible interval: 64,920–83,283) compared with 100,868 [95%CI: 58,534–143,202] in 2004. The proportion of undiagnosed individuals in 2004 was estimated at 43% but varied greatly according to age and gender. In 2004, chronically-infected women in the 18–59 age-group were undiagnosed less frequently than their male counterparts, most likely thanks to HCV testing concomitant with mandatory HBV and HIV antenatal screening. Fewer than a quarter of the 40–59 year old age-group were undiagnosed, this proportion reaching nearly three quarters among the 60–80 year old group, leading to a predominance of the eldest among those undiagnosed in 2004. One may assume that the majority of the 60–80 year old group had been infected in the distant past, most probably through blood transfusion, and did not consider themselves at risk. Indeed, almost one third of the undiagnosed HCV population in 2004 reported a history of blood transfusion before 1992. The actual proportion was probably higher since a history of blood transfusion may sometimes be unknown to patients and/or physicians.

The decision to update data for the undiagnosed HCV population for 2014 was based on our expectation that the number of undiagnosed among the 18–80 year old age-group would be substantially smaller than that in 2004. Indeed, this decision was justified as a reduction of 27% was estimated. This reduction was largely due to the decrease seen in the 60–80 year old group (approximately 50%), either because of death or because they turned 81. Almost half of the undiagnosed HCV population in 2014 was male aged between 18 and 59 years old.

This finding contributes to the recommendation of a new French complementary screening strategy included in the first French report on the management of patients infected with hepatitis C or B virus in 2014 [22]. This new recommendation promotes the expansion of HCV screening to include one-time simultaneous HCV, HBV and HIV testing in men aged 18–60, independently of risk exposure. It is based on the fact that there are similarities between these three viruses, in terms of epidemiology, transmission modes and the predominance of males in the undiagnosed populations (more than 70% for HIV in 2010 [45] and almost 80% for HBV in 2004 [22,46]). Moreover, combined testing (as opposed to using different testing strategies) for these three viruses may improve acceptability by general practitioners. The real-world application of testing recommendations by primary care physicians is of critical importance. For example, the application of the 2009-recommendation for routine HIV testing among the 15–70 year old general population in France has been poor, partly because of feasibility problems [47]. Several studies have also highlighted the poor uptake of the American recommendation for HCV birth cohort screening by physicians in the US [12,48,49].

The present work has limitations. The first involves potential participation bias in the 2004 national prevalence survey. However, the impact of such a bias on estimates is difficult to assess as it is unclear whether HCV-diagnosed individuals were less or more inclined to go to centers for a medical checkup [19,50]. The second limitation involves the simple epidemiologic model and the large number of data sources for the 2014 update. Since uncertainty was unknown for several parameters, global variance could not be easily calculated. Our alternative was to propose different scenarios adding uncertainties for parameters which had the greatest impact on estimates. We calculated a midpoint estimate and a plausible interval likely to contain the true value. Moreover, this approach was based on several assumptions. For example, pending HCV incidence estimate from ANRS-Coquelicot survey [37], we assumed, from literature, that the incidence rate in French IDUs was between 6% and 18% during the 2004–2014 period [23–30]. This rate range is consistent with a previous estimate (11%) for the North-Eastern region of France in 2000 [31]. For diagnosis, we assumed an annual increase of 1% in the proportion of chronically-infected HCV individuals aware of their status. This hypothesized 1%, used in previous models in France [16], is probably the lowest minimum, since data for the 1994 to 2004 period highlighted an increase from 24% to 56% in the proportion of those aware [21] and because HCV screening activity has continued to increase since the 2000’s [20]. This may have led to an overestimation of the number of undiagnosed individuals in 2014. To take into account the number of deaths, we used competitive mortality (except for incident cases among active IDUs). This assumption seems reasonable since severe liver disease was unlikely in this undiagnosed (and therefore asymptomatic) population. Finally, due to sparse data, we did not take into account migrations. However, if anything, this may have led to a slightly underestimation of the number of undiagnosed population in 2014, as migrants represented less than 15% of undiagnosed population in 2004 and migrant flows did not significantly change until 2014 [40].

One of the main strengths of this work is that it relies on a national survey performed on a large and randomly selected sample of 15,000 individuals, all tested for HCV and, concomitantly, interviewed about social, demographic, behavioral characteristics, at-risk exposure and awareness of HCV status [19]. An epidemiologic model enabled us to update data on the number of undiagnosed population for 2014. Another specificity of our work is that it focuses on the chronically-infected population (HCV RNA positive) which constitutes the target screening. This is unlike the large number of other studies based on anti-HCV positive populations which include both past and active infections. Finally, for the first time in France, this work provides estimates of the undiagnosed population, which are essential to help adapt testing strategies as HCV prevalence data alone are not sufficient, and may even misguide public health policy making. As illustrated in Fig 3, demographic characteristics of French chronically-infected HCV (diagnosed and undiagnosed) and undiagnosed populations were markedly different since the proportion of undiagnosed individuals was strongly associated with age and gender. The 1945–1965 birth cohort represented almost half of the chronically-infected HCV population but only a quarter of the undiagnosed population, since this birth cohort was the most diagnosed. The situation may be similar in the US where the highest diagnosis rate was also observed in the 1945–1965 birth cohort both in the general population [11] and among veterans [51].

According to our 2014 estimates, elderly undiagnosed people are numerous in France (nearly 45,000 in the 60–90 age-group). However, elderly people with HCV are rarely treated due to higher rates of side effects leading to discontinuation of therapy. The development of interferon-free regimens could change this paradigm and lead to reassess the age bracket testing recommendation [10].

## Conclusions

This original work contributed, in 2014, to recommend a new French screening strategy [22]. It may also help to feed models to assess if population-based screening would be cost-effective in a low-prevalence setting, taking feasibility into account. The latter would constitute one element of several screening strategies which include reinforcement of targeted screening and community testing outside clinical settings for hard-to-reach populations. However, improvement in the identification of HCV-infected population cannot be effective without expanding access to treatment for people diagnosed positive, notably given the perspective of the possible eradication of HCV infection in France [10].

## Supporting Information

S1 Table Estimated age-and-gender distribution of French active IDUs in 2004, ANRS Coquelicot survey. (DOC)

S2 Table Estimated age-and-gender distribution of French active IDUs in 2011, ANRS Coquelicot survey. (DOC)

S3 Table Estimated HCV seroprevalence among French active IDUs, by age-group and gender in 2004, ANRS Coquelicot survey. (DOC)

S4 Table Estimated HCV seroprevalence among French active IDUs, by age-group and gender in 2011, ANRS Coquelicot survey. (DOC)

S5 Table Estimated HCV incidence (per 100,000 person-years) in repeat blood donors by age-group, gender in France, 2004–2012. (DOC)

S6 Table Estimated HCV seroprevalence in the French general population by age-group and gender, 2004, mainland France. (DOC)

## References

[1] Hoofnagle JH, Sherker AH. Therapy for hepatitis C—the costs of success. N Engl J Med 2014;370(16):1552–3. 10.1056/NEJMe1401508 24725236

[2] Afdhal NH, Zeuzem S, Schooley RT, Thomas DL, Ward JW, Litwin AH, et al The new paradigm of hepatitis C therapy: integration of oral therapies into best practices. J Viral Hepat 2013;20(11):745–60. 10.1111/jvh.12173 24168254 PMC3886291

[3] Hellard M, Doyle JS, Sacks-Davis R, Thompson AJ, McBryde E. Eradication of hepatitis C infection: the importance of targeting people who inject drugs. Hepatology 2014;59(2):366–9. 10.1002/hep.26623 23873507 PMC4298812

[4] Martin NK, Vickerman P, Grebely J, Hellard M, Hutchinson SJ, Lima VD, et al Hepatitis C virus treatment for prevention among people who inject drugs: Modeling treatment scale-up in the age of direct-acting antivirals. Hepatology 2013;58(5):1598–609. 10.1002/hep.26431 23553643 PMC3933734

[5] Cousien A, Tran VC, Deuffic-Burban S, Dhersin JS, Yazdanpanah Y. Impact of new DAA-containing regimens on HCV transmission among injecting drug users (IDUS): a model-based analysis. The International Liver Congress 2014. J Hepatol 2014;60

[6] EASL Clinical Practice Guidelines: management of hepatitis C virus infection. J Hepatol 2014;60(2):392–420. 10.1016/j.jhep.2013.11.003 24331294

[7] Shivkumar S, Peeling R, Jafari Y, Joseph L, Pant PN. Accuracy of rapid and point-of-care screening tests for hepatitis C: a systematic review and meta-analysis. Ann Intern Med 2012;157(8):558–66. 10.7326/0003-4819-157-8-201210160-00006 23070489

[8] Stockman LJ, Guilfoye SM, Benoit AL, Vergeront JM, Davis JP. Rapid hepatitis C testing among persons at increased risk for infection—Wisconsin, 2012–2013. MMWR Morb Mortal Wkly Rep 2014;63(14):309–11. 24717818 PMC5779376

[9] Hopkins DR. Disease eradication. N Engl J Med 2013;368(1):54–63. 10.1056/NEJMra1200391 23281976

[10] Wedemeyer H, Duberg AS, Buti M, Rosenberg WM, Frankova S, Esmat G, et al Strategies to manage hepatitis C virus (HCV) disease burden. J Viral Hepat 2014;21 Suppl 1:60–89. 10.1111/jvh.12249 24713006

[11] Denniston MM, Klevens RM, McQuillan GM, Jiles RB. Awareness of infection, knowledge of hepatitis C, and medical follow-up among individuals testing positive for hepatitis C: National Health and Nutrition Examination Survey 2001–2008. Hepatology 2012;55(6):1652–61. 10.1002/hep.25556 22213025 PMC4586034

[12] Smith BD, Morgan RL, Beckett GA, Falck-Ytter Y, Holtzman D, Teo CG, et al Recommendations for the identification of chronic hepatitis C virus infection among persons born during 1945–1965. MMWR Recomm Rep 2012;61(RR-4):1–32. 22895429

[13] Grebely J, Bilodeau M, Feld JJ, Bruneau J, Fischer B, Raven JF, et al The Second Canadian Symposium on hepatitis C virus: a call to action. Can J Gastroenterol 2013;27(11):627–32. 24199209 10.1155/2013/242405 PMC3816942

[14] Agence nationale d'accréditation et d'évaluation en santé. Dépistage de l'hépatite C—Populations à dépister et modalités du dépistage. Recommandations du comité d'experts réuni par l'ANAES. 2001.

[15] Health consumer powerhouse. Euro Hepatitis Index report. 2012.

[16] Deuffic-Burban S, Deltenre P, Buti M, Stroffolini T, Parkes J, Muhlberger N, et al Predicted effects of treatment for HCV infection vary among European countries. Gastroenterology 2012;143(4):974–85. 10.1053/j.gastro.2012.05.054 22863764

[17] Bruggmann P, Berg T, Ovrehus AL, Moreno C, Brandao Mello CE, Roudot-Thoraval F, et al Historical epidemiology of hepatitis C virus (HCV) in selected countries. J Viral Hepat 2014;21 Suppl 1:5–33. 10.1111/jvh.12247 24713004

[18] Plan national de lutte contre les hépatites B et C (2009–2012). Ministère de la santé et des sports [updated 2009];Available: http://www.sante-sports.gouv.fr/IMG/pdf/Plan_national_Hepatites.pdf

[19] Meffre C, Le Strat Y, Delarocque-Astagneau E, Dubois F, Antona D, Lemasson JM, et al Prevalence of hepatitis B and hepatitis C virus infections in France in 2004: social factors are important predictors after adjusting for known risk factors. J Med Virol 2010;82(4):546–55. 10.1002/jmv.21734 20166185

[20] Brouard C, Delarocque-Astagneau E, Meffre C, Pioche C, Silvain C, Larsen C, et al Evolution du dépistage de l'hépatite C en France à partir des systèmes de surveillance RENA-VHC et des pôles de référence, 2000–2007. Bull Epidémiol Hebd 2009;20–21:199–204. 10.3945/jn.111.146258 24737153

[21] Delarocque-Astagneau E, Meffre C, Dubois F, Pioche C, Le Strat Y., Roudot-Thoraval F, et al The impact of the prevention programme of hepatitis C over more than a decade: the French experience. J Viral Hepat 2010;17(6):435–43. 10.1111/j.1365-2893.2009.01196.x 19780936

[22] Prise en charge des personnes infectées par les virus de l'hépatite B ou de l'hépatite C. Rapport de recommandations 2014. Sous la direction du Professeur Daniel Dhumeaux et sous l'égide de l'ANRS et de l'AFEF. 2014.

[23] van den Berg CH, Smit C, Bakker M, Geskus RB, Berkhout B, Jurriaans S, et al Major decline of hepatitis C virus incidence rate over two decades in a cohort of drug users. Eur J Epidemiol 2007;22(3):183–93. 17334821 10.1007/s10654-006-9089-7 PMC2781102

[24] Brant LJ, Ramsay ME, Balogun MA, Boxall E, Hale A, Hurrelle M, et al Diagnosis of acute hepatitis C virus infection and estimated incidence in low- and high-risk English populations. J Viral Hepat 2008;15(12):871–7. 10.1111/j.1365-2893.2008.01009.x 18637073

[25] Hope VD, Hickman M, Ngui SL, Jones S, Telfer M, Bizzarri M, et al Measuring the incidence, prevalence and genetic relatedness of hepatitis C infections among a community recruited sample of injecting drug users, using dried blood spots. J Viral Hepat 2011;18(4):262–70. 10.1111/j.1365-2893.2010.01297.x 20456636

[26] Craine N, Hickman M, Parry JV, Smith J, Walker AM, Russell D, et al Incidence of hepatitis C in drug injectors: the role of homelessness, opiate substitution treatment, equipment sharing, and community size. Epidemiol Infect 2009;137(9):1255–65. 10.1017/S095026880900212X 19224654

[27] Hagan H, Pouget ER, Williams IT, Garfein RL, Strathdee SA, Hudson SM, et al Attribution of hepatitis C virus seroconversion risk in young injection drug users in 5 US cities. J Infect Dis 2010;201(3):378–85. 10.1086/649783 20053137

[28] Grebely J, Lima VD, Marshall BD, Milloy MJ, DeBeck K, Montaner J, et al Declining Incidence of Hepatitis C Virus Infection among People Who Inject Drugs in a Canadian Setting, 1996–2012. PLoS One 2014;9(6):e97726 10.1371/journal.pone.0097726 24897109 PMC4045728

[29] Wandeler G, Gsponer T, Bregenzer A, Gunthard HF, Clerc O, Calmy A, et al Hepatitis C virus infections in the Swiss HIV Cohort Study: a rapidly evolving epidemic. Clin Infect Dis 2012;55(10):1408–16. 10.1093/cid/cis694 22893583

[30] Page-Shafer K, Pappalardo BL, Tobler LH, Phelps BH, Edlin BR, Moss AR, et al Testing strategy to identify cases of acute hepatitis C virus (HCV) infection and to project HCV incidence rates. J Clin Microbiol 2008;46(2):499–506. 18032621 10.1128/JCM.01229-07 PMC2238141

[31] Lucidarme D, Bruandet A, Ilef D, Harbonnier J, Jacob C, Decoster A, et al Incidence and risk factors of HCV and HIV infections in a cohort of intravenous drug users in the North and East of France. Epidemiol Infect 2004;132(4):699–708. 15310172 10.1017/s095026880400247x PMC2870151

[32] Grebely J, Matthews GV, Lloyd AR, Dore GJ. Elimination of hepatitis C virus infection among people who inject drugs through treatment as prevention: feasibility and future requirements. Clin Infect Dis 2013;57(7):1014–20. 10.1093/cid/cit377 23728143

[33] Valdiserri R, Khalsa J, Dan C, Holmberg S, Zibbell J, Holtzman D, et al Confronting the emerging epidemic of HCV infection among young injection drug users. Am J Public Health 2014;104(5):816–21. 10.2105/AJPH.2013.301812 24625174 PMC3987598

[34] Costes JM. Prévalence de l'usage problématique de drogues en France: estimations 2006. Saint-Denis: Office Français des Drogues et des Toxicomanies; 2009.

[35] Janssen E, Bastie A. Usage problématique de drogues en France: les prévalences en 2011. Saint-Denis: Office français des drogues et des toxicomanies; 2013.

[36] Jauffret-Roustide M, Le Strat Y, Couturier E, Thierry D, Rondy M, Quaglia M, et al A national cross-sectional study among drug-users in France: epidemiology of HCV and highlight on practical and statistical aspects of the design. BMC Infect Dis 2009;9:113 10.1186/1471-2334-9-113 19607712 PMC2733898

[37] Jauffret RM, Pillonel J, Weill BL, Leon L, Le Strat Y, Brunet S, et al Estimation de la séroprévalence du VIH et de l'hépatite C chez les usagers de drogues en France. Premiers résultats de l'enquête ANRS-Coquelicot 2011. Bull Epidemiol Hebd 2013;(39–40):504–9.

[38] Amin J, Law MG, Micallef J, Jauncey M, VAN B, I, Kaldor JM, et al Potential biases in estimates of hepatitis C RNA clearance in newly acquired hepatitis C infection among a cohort of injecting drug users. Epidemiol Infect 2006;1–7. 16707030 10.1017/S0950268806006388 PMC2870537

[39] Laperche S, Servant DA, Gallian P, Pillonel J. La surveillance de la diversité des virus VIH, VHB et VHC chez les donneurs de sang français entre 2000 et 2010. Bull Epidemiol Hebd 2012;(39–40):447–52.

[40] National Institute of Statistics and Economic Studies (INSEE). http://wwwinseefr/en [updated 2014];

[41] Pillonel J, Laperche S. Trends in risk of transfusion-transmitted viral infections (HIV, HCV, HBV) in France between 1992 and 2003 and impact of nucleic acid testing (NAT). Euro Surveill 2005;10(2):5–8. 15735313 10.2807/esm.10.02.00519-en

[42] Lopez D, Martineau H, Palle C. Mortalité liée aux drogues illicites. Etude d'une cohorte rétrospective de personnes interpellées pour usage de stupéfiants. Saint-Denis: Office français des drogues et des toxicomanies; 2004.

[43] Kielland KB, Skaug K, Amundsen EJ, Dalgard O. All-cause and liver-related mortality in hepatitis C infected drug users followed for 33 years: a controlled study. J Hepatol 2013;58(1):31–7. 10.1016/j.jhep.2012.08.024 22960427

[44] World Health Organization. Global distribution of hepatitis A, B and C, 2001. Weekly epidemiological record 2002;77:41–8.

[45] Supervie V, Ndawinz JD, Lodi S, Costagliola D. The undiagnosed HIV epidemic in France and its implications for HIV screening strategies. AIDS 2014;28(12):1797–804. 10.1097/QAD.0000000000000270 24681416 PMC4262966

[46] Brouard C, Pillonel J, Le Strat Y, Jauffret-Roustide M, Lot F, Larsen C, et al Characteristics of undiagnosed HBV or HCV chronically infected population in France: a need for reconsidering testing. The International Liver Congress 2014. J Hepatol 2014;60

[47] Yazdanpanah Y, Champenois K. Assessing characteristics of hidden epidemics to design the most efficient HIV testing strategies. AIDS 2014;28(12):1831–3. 10.1097/QAD.0000000000000271 25006827

[48] Albers C, Qamar A, Tellier M, Gordon F. Hepatitis C screening rates at a single center after the release of a CDC Recommendations to screen all adults born between 1945 and 1965. Hepatology 2013;58(S1):917A.

[49] Geboy A, Mahajan S, Fleming I, Daly A, Sewell C, Cole C. Hepatitis C virus birth cohort testing and linkage to care (HepTLC) in a large Washington DC medical center. Hepatology 2013;58(S1):1290A.

[50] Cazein F, Barin F, Le Strat Y, Pillonel J, LeVu S, Lot F, et al Prevalence and characteristics of individuals with undiagnosed HIV infection in France: evidence from a survey on hepatitis B and C seroprevalence. J Acquir Immune Defic Syndr 2012;60(4):e114–e117. 10.1097/QAI.0b013e318256b3fd 22772350

[51] Backus LI, Belperio PS, Loomis TP, Mole LA. Impact of Race/Ethnicity and Gender on HCV Screening and Prevalence Among US Veterans in Department of Veterans Affairs Care. Am J Public Health 2014;104 Suppl 4:S555–S561. 10.2105/AJPH.2014.302090 25100421 PMC4151890

