# Supplementary material for: The Undiagnosed Chronically-Infected HCV Population in France. Implications for Expanded Testing Recommendations in 2014
Source: PLoS One. 2015 May 11;10(5):e0126920. doi: 10.1371/journal.pone.0126920 (PMC4427442; doi:10.1371/journal.pone.0126920)
Supplement: S1 Table — (DOC) [file pone.0126920.s001.doc]

**S1 Table: Estimated age-and-gender distribution of French active IDUs in 2004, ANRS Coquelicot survey [1]**

| |  |  |  | | --- | --- | --- | | Men | Women |
| --- | --- | --- | --- | --- | --- |
| 18-29 | 26% | 34% |
| 30-39 | 53% | 44% |
| 40-49 | 21% | 22% |
| 50-59 | 0% | 0% |
| 60-69 | 0% | 0% |
| 70-80 | 0% | 0% |

Supplementary references

[1] Jauffret-Roustide M, Le Strat Y, Couturier E, Thierry D, Rondy M, Quaglia M, *et al*. A national cross-sectional study among drug-users in France: epidemiology of HCV and highlight on practical and statistical aspects of the design. BMC Infect Dis 2009;9:113.
