# Supplementary material for: The Undiagnosed Chronically-Infected HCV Population in France. Implications for Expanded Testing Recommendations in 2014
Source: PLoS One. 2015 May 11;10(5):e0126920. doi: 10.1371/journal.pone.0126920 (PMC4427442; doi:10.1371/journal.pone.0126920)
Supplement: S2 Table — (DOC) [file pone.0126920.s002.doc]

**S2 Table: Estimated age-and-gender distribution of French active IDUs in 2011, ANRS Coquelicot survey [1]**

| |  |  |  | | --- | --- | --- | | Men | Women |
| --- | --- | --- | --- | --- | --- |
| 18-29 | 17% | 32% |
| 30-39 | 43% | 40% |
| 40-49 | 34% | 25% |
| 50-59 | 6% | 3% |
| 60-69 | 0% | 0% |
| 70-80 | 0% | 0% |

Supplementary references

[1] Jauffret RM, Pillonel J, Weill BL, Leon L, Le Strat Y, Brunet S, *et al*. Estimation de la séroprévalence du VIH et de l'hépatite C chez les usagers de drogues en France . Premiers résultats de l'enquête ANRS-Coquelicot 2011. Bull Epidemiol Hebd 2013;(39-40):504-9.
