# Supplementary material for: The Undiagnosed Chronically-Infected HCV Population in France. Implications for Expanded Testing Recommendations in 2014
Source: PLoS One. 2015 May 11;10(5):e0126920. doi: 10.1371/journal.pone.0126920 (PMC4427442; doi:10.1371/journal.pone.0126920)
Supplement: S3 Table — (DOC) [file pone.0126920.s003.doc]

**S3 Table: Estimated HCV seroprevalence among French active IDUs, by age-group and gender in 2004, ANRS Coquelicot survey [1]**

|  | Men | Women |
| --- | --- | --- |
| < 30 | 29% | 78% |
| 30-34 | 89% | 87% |
| 35-39 | 90% | 81% |
| 40 and more | 91% | 84% |

Supplementary references

[1] Jauffret-Roustide M, Le Strat Y, Couturier E, Thierry D, Rondy M, Quaglia M, *et al*. A national cross-sectional study among drug-users in France: epidemiology of HCV and highlight on practical and statistical aspects of the design. BMC Infect Dis 2009;9:113.
