# Supplementary material for: The Undiagnosed Chronically-Infected HCV Population in France. Implications for Expanded Testing Recommendations in 2014
Source: PLoS One. 2015 May 11;10(5):e0126920. doi: 10.1371/journal.pone.0126920 (PMC4427442; doi:10.1371/journal.pone.0126920)
Supplement: S4 Table — (DOC) [file pone.0126920.s004.doc]

**S4 Table: Estimated HCV seroprevalence among French active IDUs, by age-group and gender in 2011, ANRS Coquelicot survey [1]**

|  | Men | Women |
| --- | --- | --- |
| < 30 | 24% | 31% |
| 30-34 | 59% | 31% |
| 35-39 | 65% | 92% |
| 40 and more | 92% | 83% |

Supplementary references

[1] Jauffret RM, Pillonel J, Weill BL, Leon L, Le Strat Y, Brunet S, *et al*. Estimation de la séroprévalence du VIH et de l'hépatite C chez les usagers de drogues en France . Premiers résultats de l'enquête ANRS-Coquelicot 2011. Bull Epidemiol Hebd 2013;(39-40):504-9.
