# Supplementary material for: The Undiagnosed Chronically-Infected HCV Population in France. Implications for Expanded Testing Recommendations in 2014
Source: PLoS One. 2015 May 11;10(5):e0126920. doi: 10.1371/journal.pone.0126920 (PMC4427442; doi:10.1371/journal.pone.0126920)
Supplement: S5 Table — (DOC) [file pone.0126920.s005.doc]

**S5 Table: Estimated HCV incidence (per 100,000 person-years) in repeat blood donors by age-group, gender in France, 2004-2012 (method described in [1])**

|  | Men | Women |
| --- | --- | --- |
| 18-29 | 0.13 | 0.12 |
| 30-39 | 0.41 | 0.75 |
| 40-49 | 0.20 | 0.51 |
| 50-65* | 0.23 | 0.28 |

** 50-70 since 2010*

Supplementary references

[1] Pillonel J, Laperche S. Trends in risk of transfusion-transmitted viral infections (HIV, HCV, HBV) in France between 1992 and 2003 and impact of nucleic acid testing (NAT). Euro Surveill 2005;10(2):5-8.
