# Supplementary material for: The Undiagnosed Chronically-Infected HCV Population in France. Implications for Expanded Testing Recommendations in 2014
Source: PLoS One. 2015 May 11;10(5):e0126920. doi: 10.1371/journal.pone.0126920 (PMC4427442; doi:10.1371/journal.pone.0126920)
Supplement: S6 Table — (DOC) [file pone.0126920.s006.doc]

**S6 Table: Estimated HCV seroprevalence in the French general population by age-group and gender, 2004, mainland France [1]**

|  | Men | Women |
| --- | --- | --- |
| 18-29 | 0.13% | 0.03% |
| 30-39 | 0.74% | 0.29% |
| 40-49 | 1.12% | 2.15% |
| 50-59 | 0.29% | 0.80% |
| 60-69 | 0.85% | 2.22% |
| 70-80 | 1.13% | 1.10% |

Supplementary references

[1] Meffre C, Le Strat Y, Delarocque-Astagneau E, Dubois F, Antona D, Lemasson JM, *et al*. Prevalence of hepatitis B and hepatitis C virus infections in France in 2004: social factors are important predictors after adjusting for known risk factors. J Med Virol 2010;82(4):546-55.
